# Creep and Recovery Behaviour of Polyolefin-Rubber Nanocomposites Developed for Additive Manufacturing

**DOI:** 10.3390/polym8120437

**Published:** 2016-12-15

**Authors:** Fugen Daver, Mladenko Kajtaz, Milan Brandt, Robert A. Shanks

**Affiliations:** 1 School of Engineering, RMIT University, P.O. Box 71, Bundoora, Victoria 3083, Australia; mladenko.kajtaz@rmit.edu.au (M.K.); milan.brandt@rmit.edu.au (M.B.); 2 School of Science, RMIT University, GPO Box 2476, Melbourne, Victoria 3001, Australia; robert.shanks@rmit.edu.au

**Keywords:** polyethylene, polymer-matrix composite, creep and recovery, modelling, nanocomposite

## Abstract

Nanocomposite application in automotive engineering materials is subject to continual stress fields together with recovery periods, under extremes of temperature variations. The aim is to prepare and characterize polyolefin-rubber nanocomposites developed for additive manufacturing in terms of their time-dependent deformation behaviour as revealed in creep-recovery experiments. The composites consisted of linear low density polyethylene and functionalized rubber particles. Maleic anhydride compatibilizer grafted to polyethylene was used to enhance adhesion between the polyethylene and rubber; and multi-walled carbon nanotubes were introduced to impart electrical conductivity. Various compositions of nanocomposites were tested under constant stress in creep and recovery. A four-element mechanistic Burger model was employed to model the creep phase of the composites, while a Weibull distribution function was employed to model the recovery phase of the composites. Finite element analysis using Abaqus enabled numerical modelling of the creep phase of the composites. Both analytical and numerical solutions were found to be consistent with the experimental results. Creep and recovery were dependent on: (i) composite composition; (ii) compatibilizers content; (iii) carbon nanotubes that formed a percolation network.

## 1. Introduction

Dimensional stability of composites over their expected lifetime is an important aspect for engineering applications. This is particularly important when the innovative functional polymeric materials incorporating nanomaterials are used in integrated systems such as automotive applications and the components are exposed to varying stresses over extended periods at elevated temperatures. Creep and recovery experiments present a sensitive means of assessing the dimensional stability of polymeric materials by understanding their viscoelastic deformation behaviour under constant stress and temperature [1,2,3,4]. 

In creep experiment, a constant stress (σ) is applied instantaneously to a material under isothermal conditions, and the resulting strain (ε) is measured as a function of time. Total strain consists of (i) reversible and recoverable elastic strain (ε_e_); (ii) time-dependent viscoelastic strain (ε_ve_); and (iii) inelastic viscous strain (ε_v_). The material first experiences an instantaneous, elastic deformation upon application of the stress. Viscous and viscoelastic flow then occur at a decreasing rate before the deformation reaches a steady state. An instantaneous, elastic recovery takes place with a sharp decrease in strain upon removal of the stress. Time dependent viscoelastic recovery proceeds at a slower rate. The strain remaining at the end of the test indicates permanent viscous flow that is irrecoverable and it is referred to as unrecovered creep or the permanent set of the material. 

Creep behaviour is a time-temperature dependent phenomenon and various theoretical and mechanistic models have been used to describe viscoelastic behaviour of polymeric materials [5]. In linear viscoelastic behaviour, at any fixed time (isochronous) following initiation of a loading history, the strain should be proportional to the stress and the strain generated by a load should be independent from any previously applied load. Otherwise, the limits of linearity will be reached and the material can no longer be considered as linear viscoelastic. The degree of non-linearity can be influenced by factors such as applied stress level, strain rate, and temperature. All linear viscoelastic models to define creep behaviour are made up of linear springs and linear dashpots [6]. 

For solids that exhibit only elastic behaviour, the material is represented by an ideal spring element, where *E* is the Hookean elastic modulus (Equation (1)). The implication is that the energy stored is recoverable and this translates to the immediate restoration of the original state upon removal of the stress.

(1) σ=Eε

A material is represented by a dashpot when it exhibits viscous liquid behaviour, where η is the Newtonian viscosity (Equation (2)). The viscous deformation resulting from an applied stress is a time-dependent response. The deformation is linear with time under a constant stress and the imposed deformation is permanent.

(2) $$\sigma =\eta \frac{d\epsilon }{dt}$$

The Maxwell model combines these two elements in series and assumes an additive contribution from an elastic spring and viscous dashpot (Equation (3)).

(3) $$\frac{d\epsilon }{dt}=\left(\frac{1}{E}\right)\;\left(\frac{d\sigma }{dt}\right)+\frac{\sigma }{\eta }$$

The Kelvin-Voigt model incorporates the two elements in parallel with the stress apportioned to both elements. The time dependent response of a dashpot is accompanied by the restorative force of the spring. A four element Burger model is composed of the Maxwell model and Kelvin-Voigt model in series [7]. The springs correspond to elastic sections with moduli *E*_1_ and *E*_2_ while the dashpots represent the viscosities η_1_ and η_2_. The four element Burger model has been used with varying degree of success in predicting creep behaviour of reinforced composites [8,9]. The model incorporates the elastic (ε_e_), viscous (ε_v_), and viscoelastic (ε_ve_) components of the strain to describe the mechanical creep behaviour of polymeric materials (Equation (4)).

(4) $$\epsilon \left(t\right)=\frac{\sigma }{E_{1}}+\frac{\sigma t}{\eta_{1}}+\frac{\sigma }{E_{2}}\left[1-\text{exp}\left(-\frac{E_{2}\;t}{\eta_{2}}\right)\right]$$

Retardation time, τ is obtained from the exponential term of the Equation (4) and it is a measure of the time required to reach 63.2% or $\left(1-\frac{1}{e}\right)$ of the total deformation of the material in the Kelvin-Voigt unit (Equation (5)).

(5) $$\tau =\frac{\eta_{2}}{E_{2}}$$

The recovery behaviour of thermoplastics has been successfully modelled by means of a Weibull distribution function [10,11,12]. The Weibull distribution function enables prediction of time dependent recovery strain $(\varepsilon_{r}$) in the material in terms of viscoelastic strain recovery ($\varepsilon_{\text{ve}}$) and permanent strain ($\varepsilon_{\infty }$) upon removal of the stress as shown in Equation (6).

(6) $$\varepsilon_{r}\left(t\right)=\varepsilon_{\text{ve}}\left[\text{exp}{\left(-\frac{t-t_{0}}{\eta_{r}}\right)}^{\beta_{r}}\right]+\varepsilon_{\infty }$$

where, the $\varepsilon_{\text{ve}}$ is defined by the characteristic life factor ($\eta_{r}$) and shape parameters ($\beta_{r}$) over recovery time *t* and *t*_0_ is the time of stress removal. 

In an earlier study, we have developed polyolefin-rubber composites by melt mixing linear low density polyethylene with functionalized (i.e., de-vulcanized and surface activated) rubber particles through interactions of pre-functionalized polymer in the interface [13]. Increasing the rubber content of the composite from 30% to 70% in the overall composite composition decreased the elastic modulus and tensile strength while increasing the percentage elongation at break. Characterization of the composites in terms of their mechanical properties confirmed that the inclusion of the grafted maleic anhydride as compatibilizer in the composites enhanced the elongation at break and produced toughened polyolefin-rubber composites. Incorporation of carbon nanotubes with the help of a master-batch improved the electrical conductivity of the nanocomposites by selective localization of carbon nanotubes in the polyethylene phase [13]. 

Incorporation of rubber particles, which inherently possess elastomeric properties, is expected to have an adverse effect on the overall creep properties of the composites due to low elastic modulus of rubber phase. In this study, the creep and recovery behaviour of nanocomposites under different stresses and temperatures was modelled by means of analytical and numerical methods. The aim was to elucidate the effect of (i) composite composition; (ii) compatibilizers; and (iii) carbon nanotubes on the viscoelastic deformation of the material.

## 2. Materials and Methods

### 2.1. Materials

Linear low-density polyethylene (LLDPE) was melt mixed with maleic anhydride grafted polyethylene (MA-*g*-PE) which is grafted with maleic anhydride 0.90%·*w*/*w*. Functionalized rubber particles (FRP) that were produced according to a patented process were obtained from Polymeric Powders Company, Melbourne, Australia. Multi-walled carbon nanotubes (MWCNT) were incorporated into the polyolefin–rubber composites in the form of a masterbatch, Plasticyl LDPE2001 (Nanocyl, Sambreville, Belgium) that is based on low density polyethylene (LDPE) [14]. 

### 2.2. Composite Preparation

LLDPE was fed into the pre-heated non-intermeshing, counter rotating internal batch mixer at 180 °C and it was mixed at 50 rpm for 1 min to ensure complete melting of polymer prior to the introduction of the masterbatch Plasticyl LDPE2001, FRP, and MA-*g*-PE, to improve compatibility between the rubber and the polyethylene. All four ingredients were mixed for another 5 min at 180 °C before the mixture was removed for further processing. Table 1 shows the compositions of the LLDPE–FRP nanocomposites. The specimens for all creep and recovery experiments were prepared by compression moulding at 180 °C for 5 min under a force of 50 kN followed by water-cooling of the mould to below 50 °C prior to pressure release and ejection from the mould. Compression moulded plaques were cut into strips of approximately 25 × 4.5 × 0.5 mm dimensions. Details of the specimen preparation are given elsewhere [13].

### 2.3. Thermomechanical Properties

A Q800 Dynamic Mechanical Analyser (TA Instruments, New Castle, DE, USA) in tensile mode was used for creep- recovery (static force thermomechanometry, sf-TM). Sf-TM analysis was performed by subjecting the test specimens to an applied stress of 1.2 MPa for 30 min followed by a recovery period of 120 min at 25 °C according to ASTM standards [15]. The applied stress was chosen to be within the linear viscoelastic region of all composites tested as identified from the isochronous stress–strain curves. Tests were conducted at a series of temperatures, from room temperature to 80 °C, to obtain long term deformation behaviour of the material by employing time-temperature superposition principle. 

## 3. Results and Discussion

### 3.1. Strain Response of Composites with Creep Stress

**Figure 1:**
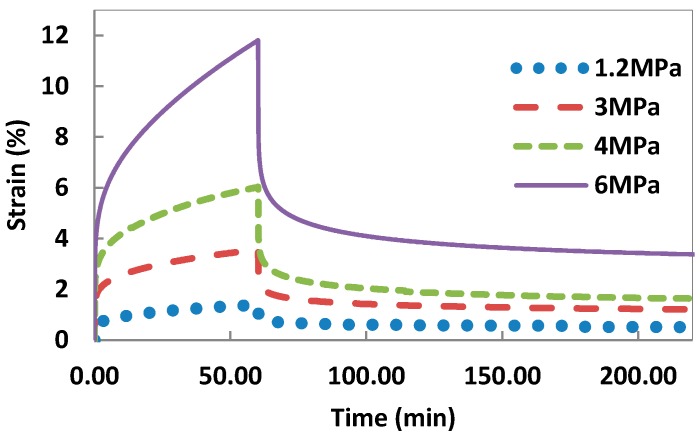
Creep-recovery response of composite material (PB1) at different initial stresses.

The creep and recovery curves of polyolefin-rubber composite (PB1) are presented in Figure 1. The creep stress varied between 1.2 and 6 MPa. In all creep curves, an instantaneous increase in strain occurs due to elastic response of the material. This is followed by a viscoelastic response, which involves time-dependent molecular rearrangement. Viscous flow is observed towards the end of the load application period. Removal of the load results in a rapid decrease in strain response, which is equal to the initial elastic response. The recovery period involves time-dependent molecular relaxation as the polymer attempts to regain original dimensions. Since the polymer experienced viscous flow, full recovery is not reached resulting in permanent deformation. Higher imposed stresses resulted in creep becoming increasingly irreversible. This permanent strain is of significant importance in engineering applications as it may result in a loss of dimensional stability under long term loading.

**Figure 2:**
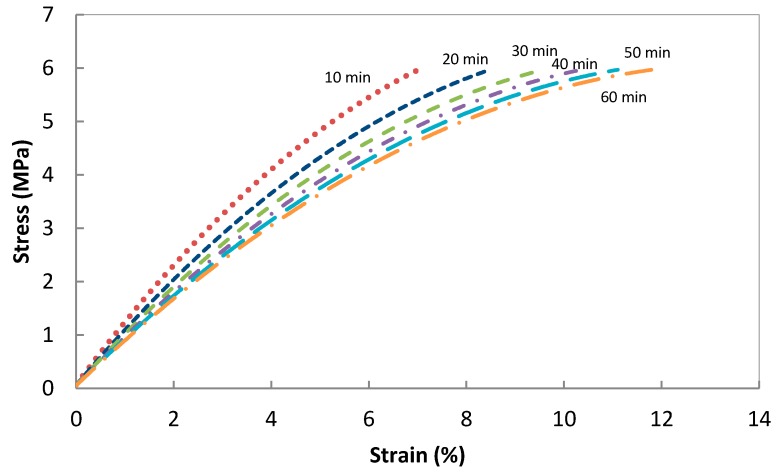
Isochronous curves obtained from creep curves as shown in Figure 1.

Isochronous curves obtained from Figure 1 are shown in Figure 2. A linear relationship between stress and strain was observed to a stress of 3 MPa. The maximum creep stress was kept below this limit so that the composites were tested within their linear viscoelastic region during creep experiments.

**Figure 3:**
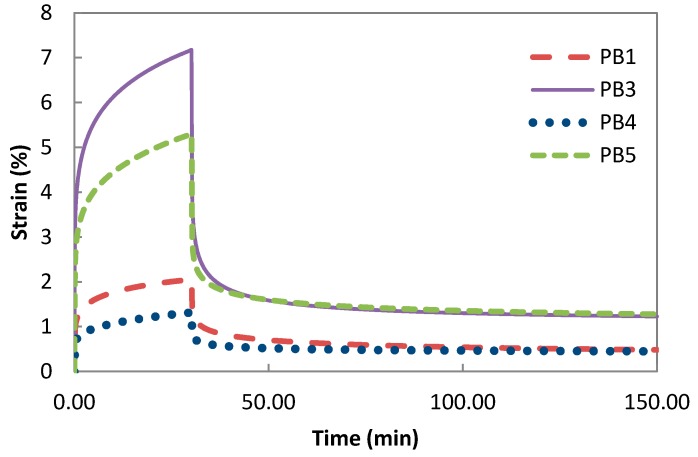
The effect of compatibilizer (MA-*g*-PE) on creep-recovery behaviour.

The creep and recovery curves for the composites PB1, PB3, PB4, and PB5 are shown in Figure 3. The addition of (MA-*g*-PE) proved to enhance the bonding between the phases and the creep deformation of PB4 and PB5 under the same load was significantly lower than the composites PB1 and PB3 which were formed without compatibilizer (MA-*g*-PE). 

**Figure 4:**
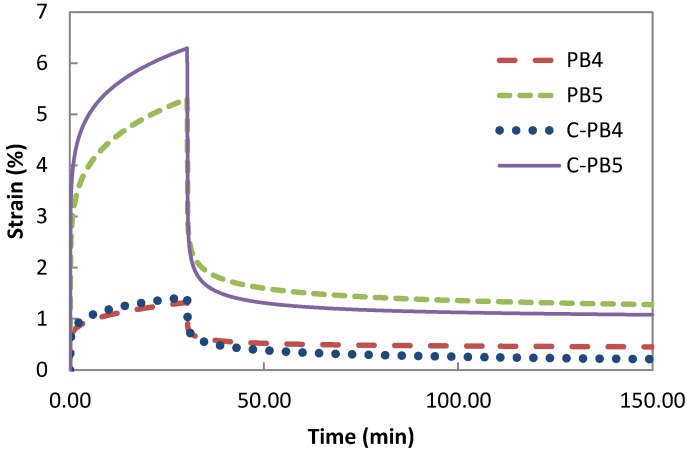
MWCNT and creep-recovery behaviour.

The creep and recovery curves for the composites PB4, C-PB4 and PB5, C-PB5 are shown in Figure 4. Incorporation of MWCNT into the composites did not introduce a constraint on deformation during the creep phase. Composites with carbon nanotubes demonstrated higher creep deformation compared with their counterpart without the carbon nanotubes. This is due to the carbon nanotubes being introduced via a low viscosity master-batch, Plasticyl LDPE2001. This finding confirms our earlier observation in regard to the decrease in Young modulus following the addition of carbon nanotubes into the composite [13]. 

### 3.2. Application of Time-Temperature Superposition for Long Term Deformation Behaviour 

**Figure 5:**
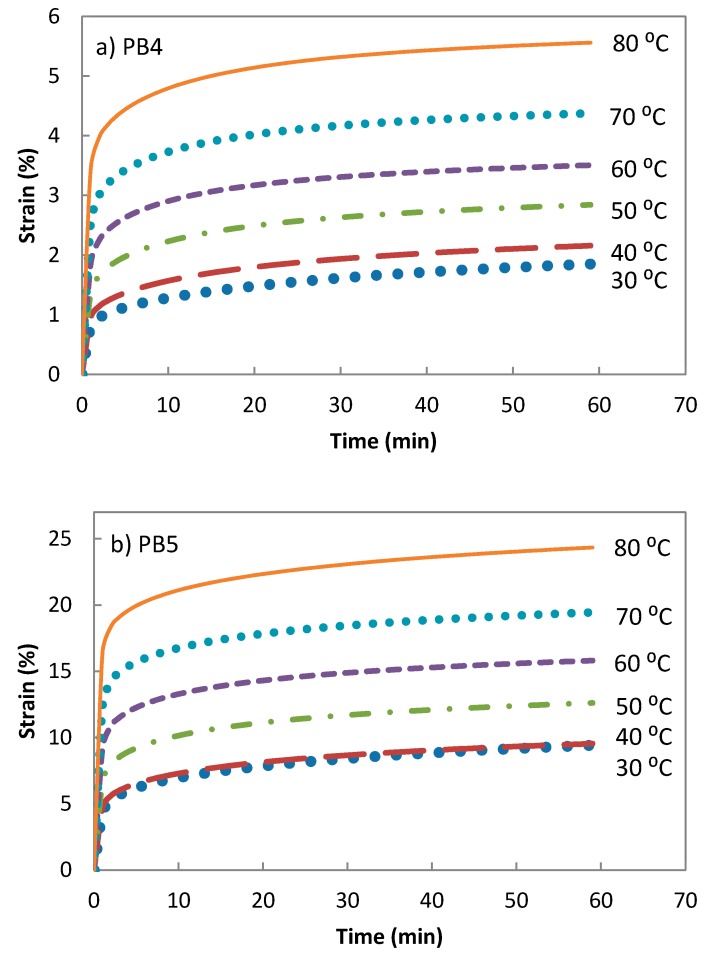
Creep response of (**a**) PB4; and (**b**) PB5 at an applied stress of 1.2 MPa.

A series of creep experiments were conducted at different temperatures, from 30 to 80 °C with 10 °C increments for the PB4 and PB5, to enable assessment of the deformation behaviour of the composites under ambient temperatures for long periods of time (Figure 5). 

Time temperature superposition principle was applied to estimate creep performance of the composite at longer durations. The relative creep-time master curves for composite PB4 and PB5 were obtained by shifting the creep strain at different temperatures, while keeping 30 °C as the reference temperature. 

The time-temperature shift factor, *a*_T_, was calculated from the shifting procedure. If an arbitrary reference temperature *T*_s_ is taken to fix one curve, then if *t*_s_ is the time of a value on the curve at *T*_s_ with a particular strain and *t* is the time of a value with the same relative creep on a curve at a different temperature, then the amount of shift required to superpose the axis is a displacement of (log *t*_s_ – log *t*) along the log time axis. The shift factor is defined by Equation (7).

(7) $$\text{log}\;a_{T}=\text{log}\;t_{s}-\text{log}\;t=\text{log}\;\frac{t_{s}}{t}$$

**Figure 6:**
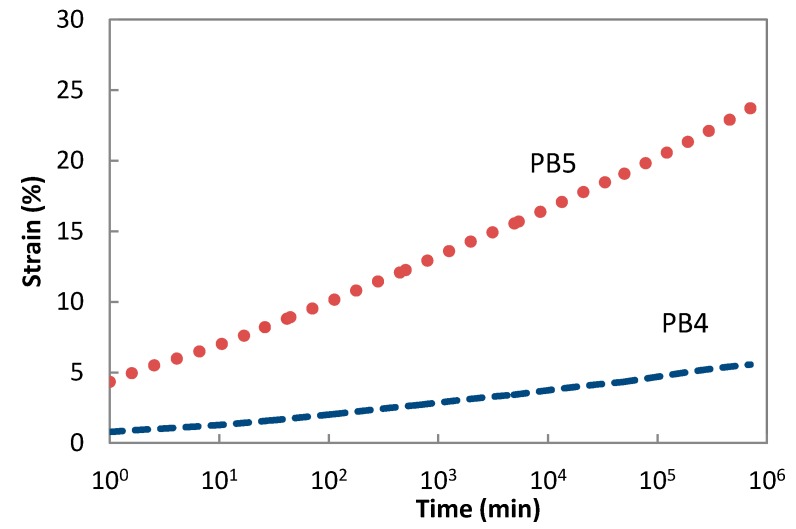
Master creep curves of PB4 and PB5 at the reference temperature 30 °C.

Figure 6 shows that creep master curves for PB4 and PB5 demonstrate a similar trend. The PB4 having a low rubber content of 30% exhibits a strain of just over 5%, while PB5 with a higher rubber content of 70% reached close to 25% strain in 1.8 y duration. 

### 3.3. Modelling of the Creep Phase of Composites via a Four Element Burger Model

The four element Burger model comprising Maxwell and Kelvin-Voigt models was used to interpret the creep component [10]. Mechanistic model parameters were extracted from creep and recovery curves of each composite (Table 2). Modulus (*E*_1_) and viscosity (η_1_) were determined from the equilibrium region of the creep curves. *E*_1_ was measured by extrapolation of the linear portion of the creep curve to the time when the stress was applied, with the modulus determined from the intercept. Increasing rubber content of the composite reduced the modulus significantly. Modulus, *E*_1_ of PB3 with 70% rubber particles (39 MPa) is much lower than the modulus of PB1 having a rubber content of 70% (192 MPa). Introduction of compatabiliser increased the modulus: PB4 and PB5 showed higher moduli compared with the PB1 and PB3, respectively. It is expected that the compatabiliser (MA-*g*-PE) should assist in promoting the connectivity of adjoining PE crystallites as well as interacting with the rubber particles. Whereas introduction of carbon nanotubes did not improve the elastic modulus, both C-PB4 and C-PB5 showed slightly reduced moduli compared with the PB4 and PB5 respectively. The equilibrium viscosity (as determined from the slope of the linear portion) accounted for the solid state viscous flow that occurred with the applied stress. C-PB4 showed the highest equilibrium viscosity, (η_1_) indicating that a lower flow occurred in the dashpot and permanent deformation decreased as shown in Figure 6. C-PB4 composite with low rubber content reinforced with carbon nanotubes is likely to be more dimensionally stable than the others when under stress. Retardation time, (τ) is equal to the ratio of the viscosity, (η_2_) to the modulus (*E*_2_) of the Kelvin-Voigt component. The non-linear curve fit function of Excel software was employed to define the modulus (*E*_2_), and the viscosity (η_2_). The modulus (*E*_2_), which is related to the stiffness of the amorphous chains in short term, increased following the incorporation of compatabiliser into the composites. Both PB4 and PB5 showed a higher modulus (*E*_2_) compared with the PB1 and PB3 respectively. The modulus (*E*_2_) of C-PB4 and C-PB5 decreased compared with the PB4 and PB5 respectively. The retardation time (τ) and the viscosity (η_2_) increased both for C-PB4 and C-PB5 compared to PB4 and PB5, respectively. Incorporation of carbon nanotubes did not introduce reinforcement in the Kelvin-Voigt model, due to low viscosity of the master batch.

### 3.4. ABAQUS Implementation of the Four Element Burger Model

In ABAQUS, creep behaviour is specified by the equivalent uniaxial behaviour—the creep law. Five common creep laws are directly provided in ABAQUS: the power law, the hyperbolic-sine law, the double power law, the Anand law, and the Darveaux law and they are used for modelling secondary or steady-state creep [16]. However, in practical cases creep laws are typically of very complex form to fit experimental data; thus, the laws are frequently user-defined via the use of user subroutine CREEP and included in a general time-dependent, viscoplastic material formulation [17,18,19]. Our four element model or Burger model, is not a standard model in ABAQUS and hence needed to be incorporated via the CREEP user subroutine. 

As the CREEP user subroutine defines the increments of inelastic creep strain; ∆ε_creep_ as functions of the solution dependent variables, such as the deviatoric stress, pressure, and temperature; and time increment ∆*t*. Equation (4) is required to be discretised to fit available integration schemes. ABAQUS provides both explicit and implicit time integration of creep and the choice of the time integration scheme depends on the procedure type, the procedure definition and a geometric non-linearity. The selected approach was based on the creep strain rate definition, shown in Equation (8), [20] and it was discretised to fit the both integration schemes.

(8) $$\dot{\epsilon }\;\left(t\right)=\frac{\sigma }{\eta_{1}}+\frac{\sigma }{\eta_{2}}\;\text{exp}\left(-\frac{t}{\tau }\right)$$

The following FORTRAN code snippet highlights some of the key aspects of the model implementation in ABAQUS.


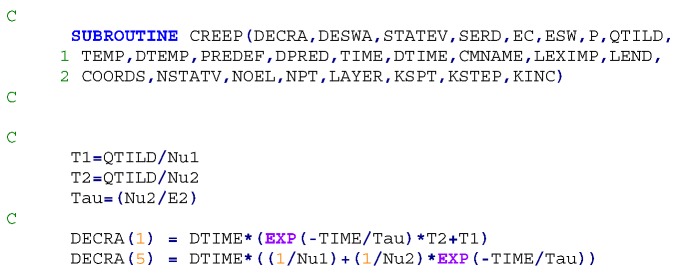


**Figure 7:**
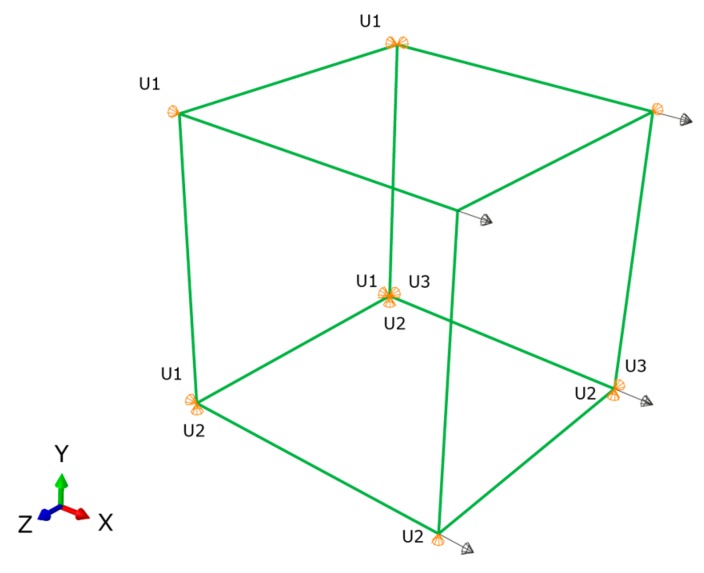
One-solid-element finite element model used in the validation.

**Figure 8:**
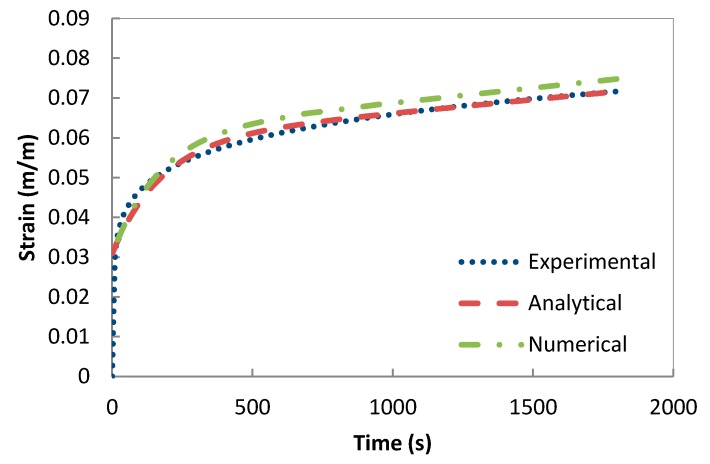
Modelling the creep component of PB3 by a Burger model.

**Figure 9:**
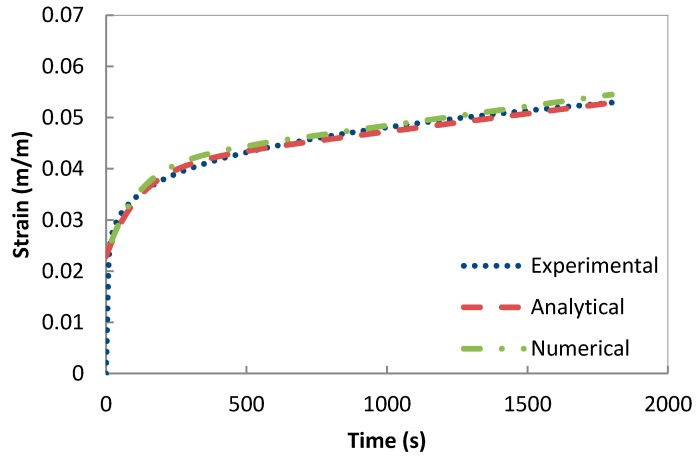
Modelling the creep component of PB5 by a Burger model.

**Figure 10:**
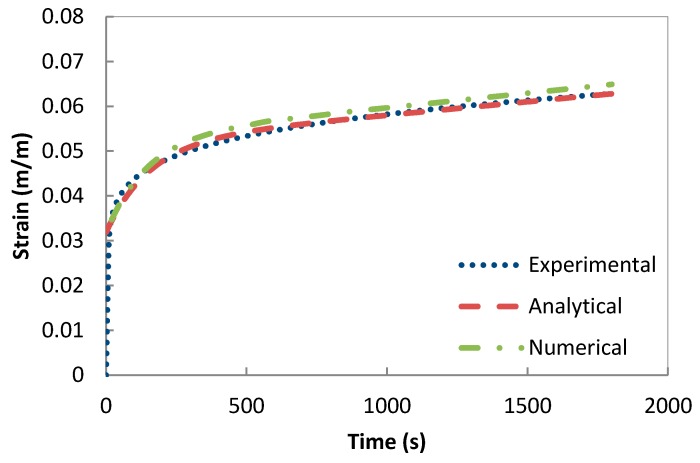
Modelling the creep component of C-PB5 by a Burger model.

The validation of the developed creep model and the previously extracted parameters was performed using a one-solid-element model shown in Figure 7 [21]. The correlation between the analytical creep strain, numerical creep strain obtained using ABAQUS Finite Element Analysis and the experimental creep strain was found to be adequate. Hence, to avoid repetition, the results were shown only for the low rubber content materials which have demonstrated higher conductivity compared the low rubber content composites upon introduction of carbon nanotubes. Figure 8, Figure 9 and Figure 10 demonstrate the consistency of both analytical and numerical solutions with the experimental results for rubber rich composites: PB3, PB5, and C-PB5 respectively. 

### 3.5. Modelling of the Recovery Phase of Composites via a Weibull Distribution Function

A Weibull distribution function was used to model the recovery component of the experimental creep curves. The value of viscoelastic strain recovery (ε_ve_) and permanent strain (ε_∞_) are received through the simulation of Weibull equation (Equation (6)) and the ε_max_ is the maximum deformation corresponding to the strain value for the longest time ($t_{0}=1800\;s$) during the creep test as shown in Equation (9).

(9) $$\varepsilon_{\text{max}}=\varepsilon_{e}+\varepsilon_{\text{ve}}+\varepsilon_{\infty }$$

**Figure 11:**
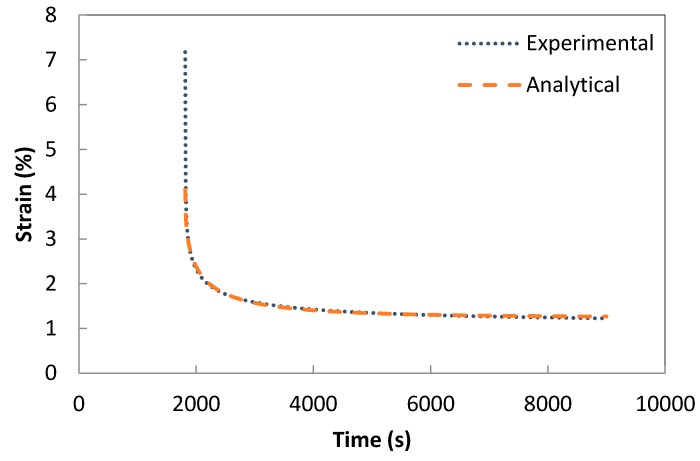
Modelling the recovery component of PB3 by a Weibull distribution function.

**Figure 12:**
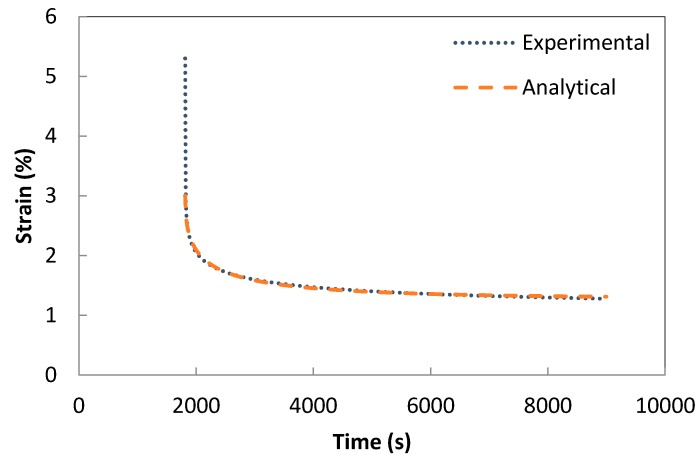
Modelling the recovery component of PB5 by a Weibull distribution function.

**Figure 13:**
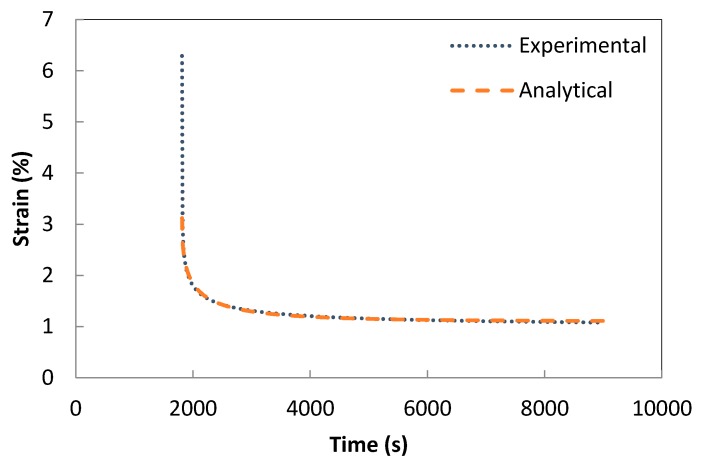
Modelling the recovery component of C-PB5 by a Weibull distribution function.

Resultant parameters are shown in Table 3. Figure 11, Figure 12 and Figure 13 show the modelling of the recovery phase of rubber rich composites—PB3, PB5, and C-PB5 respectively—by means of a Weibull distribution function. Viscoelastic strain recovery (ε_ve_) decreased from 0.95% to 0.40% for low rubber content composites (PB1 and PB4) and similarly ε_ve_ it decreased from 2.85% to 1.73% for high rubber content (PB3 and PB5) composites with the addition of the compatibilizer. This observation confirms the enhanced recovery behaviour of the composites with the addition of compatibilizers. Viscoelastic strain recovery (ε_ve_) increased from 0.40% to 0.68% for low rubber content composites (PB4 and C-PB4) and similarly ε_ve_ increased from 1.73% up to 2.02% for high rubber content composites (PB5 and C-PB5) with the addition of the carbon nanotubes. Hence, it can be concluded that the addition of carbon nanotubes did not introduce an enhanced recovery behaviour. Similarly, characteristic life factor (η_r_) and shape parameter (β_r_) are expected to show an increasing trend with the introduction of MWCNT by inhibiting the slippage of molecular chains hence leading to an increase in viscosity. However, in our studies, this trend was not clear, again most likely due to the introduction of MWCNT via a low viscosity master batch. 

## 4. Conclusions

Creep and recovery behaviour were evaluated for polyolefin-rubber nanocomposites developed for additive manufacturing. The creep component was successfully modelled by means of a four element Burger model. Model parameters enabled numerical modelling of the creep component of the composites by means of finite element analysis (Abaqus Software). Both the analytical and the numerical solutions were found to be consistent with experimental results. The recovery phase of the creep experiments was modelled using a Weibull distribution function. Model parameters, in particular change in viscoelastic strain (ε_ve_), was indicative of the effect of compatibilizers and the carbon nanotubes in time dependent recovery behaviour of the composites. 

## Figures and Tables

**Table 1:** Composition of the LLDPE–FRP nanocomposites.

Composite code	Composition (%·*w*/*w*)			
	LLDPE	FRP	MA-*g*-PE	MWCNT
PB1	70	30	-	-
PB3	30	70	-	-
PB4	65	30	5	-
PB5	25	70	5	-
C-PB4	62	30	5	3
C-PB5	22	70	5	3

**Table 2:** Parameters of the mechanistic Burger model, creep component of composites.

Composite code	*E*_1_ (MPa)	*E*_2_ (MPa)	η_1_ (MPa·s)	η_2_ (MPa·s)	τ (s)
PB1	192	127	448,200	8730	69
PB3	39	42	176,829	7409	176
PB4	258	299	479,916	13,287	44
PB5	52	69	169,956	8293	120
C-PB4	226	177	1,020,917	40,838	231
C-PB5	38	59	201,237	9177	154

**Table 3:** Parameters of the Weibull distribution function—recovery phase of composites.

Composite code	ε_max_ (%)	ε_e_ (%)	ε_ve_ (%)	ε_∞_ (%)	η_r_ (s)	β_r_
PB1	2.56	0.64	0.95	0.47	591.18	0.48
PB3	7.17	3.07	2.85	1.25	221.77	0.47
PB4	1.32	0.46	0.40	0.45	340.34	0.46
PB5	5.30	2.29	1.73	1.28	356.55	0.45
C-PB4	1.42	0.55	0.68	0.19	720.60	0.49
C-PB5	6.29	3.17	2.02	1.10	186.31	0.46
